# Developments in nitrous oxide capture technologies: bridging current research to clinical applications

**DOI:** 10.1111/anae.70080

**Published:** 2025-12-03

**Authors:** Simon Molisso, Ashleigh M. Chester, Elmira Baghdaran, Jennifer Waspe, Peyman Z. Moghadam

**Affiliations:** ^1^ Department of Chemical Engineering University College London London UK; ^2^ Manufacturing Futures Lab University College London London UK; ^3^ Nevill Hall Hospital, Aneurin Bevan University Health Board Abergavenny UK; ^4^ Sheffield Teaching Hospitals NHS Foundation Trust Sheffield UK

**Keywords:** maternity wards, metal–organic frameworks, nitrous oxide, volatile anaesthetic gases, volatile capture technology

## Abstract

**Introduction:**

Many inhaled anaesthetic agents are greenhouse gases. Capture technologies have been developed to prevent environmental emissions of volatile agents, but no such devices exist for nitrous oxide. Further to this, the unique societal position of the use of nitrous oxide for patients in labour means it cannot readily be substituted for alternatives. Currently, there are no mandated scavenging systems for nitrous oxide in maternity wards, resulting in not only loss to the environment, but also occupational exposure among labour ward staff, often at levels well above regulatory limits. Without a suitable analgesic alternative, and with contemporary catalytic destruction (cracking) devices for nitrous oxide relatively underutilised, more work must be done to develop capture technologies. While nitrous oxide capture for anaesthetic purposes is severely under‐researched, a wide range of literature exists for other applications, including directly from the atmosphere and from waste effluent during chemical processing.

**Methods:**

A literature search was used to identify original research articles describing adsorbents for nitrous oxide uptake. The search was limited to published articles over the last 5 years and relevance was screened by abstract review.

**Results:**

Different classes of adsorbents that could be used for nitrous oxide capture include activated charcoals, zeolites and metal–organic frameworks. We highlight their important properties and describe their key drawbacks. Recent literature was also examined and strategies in nitrous oxide capture across different industries drawn together to address the unique scenario of maternity analgesia.

**Discussion:**

Metal–organic frameworks are identified as a promising class of porous adsorbents that could be applied to a wide range of anaesthetic settings. With over 100,000 structures identified, they have a remarkable tuneability which should be further exploited in clinical settings to not only further progress towards ‘net zero’ targets but also to improve safety outcomes.

## Plain Language Summary

Nitrous oxide, commonly known as ‘laughing gas’, is the painkilling medication in ‘gas and air’ which is often given for pain relief in childbirth. Unfortunately, nitrous oxide is a greenhouse gas which is harmful to the environment when released into the air and contributes to climate change. It may also be harmful to midwives and other hospital staff if they breathe in too much of it. One way to stop the release of gas is to develop materials such as metal–organic frameworks that act like ‘sponges’ to trap nitrous oxide so it can be recycled or destroyed. Different sponge‐like materials have been examined to see how good they are at catching nitrous oxide. Some of these materials have never been used before in hospitals, but experiments by scientists show that they could work very well. The pros and cons of the materials are explored in this review, and the evidence suggests that metal–organic frameworks would be excellent at trapping nitrous oxide in hospital settings. Metal–organic frameworks were recently recognised with the 2025 Nobel Prize in Chemistry, with the materials unlocking new possibilities for gas capture and environmental solutions.

## Introduction

In recent years, increased awareness of the negative environmental impact of inhalational anaesthetic agents, including volatile anaesthetic agents and nitrous oxide, has motivated many anaesthetists to modify their practice, placing higher priority on good resource stewardship and sustainability [1, 2, 3, 4]. However, barriers to sustainable clinical practice are ever‐present, including limitations of hospital infrastructure, continued debate into the relative benefits of anaesthetic techniques [5, 6] and disagreement over the scale of the problem [7]. All of these risk diverting attention away from solution‐based thinking and technological innovation, which are necessary to reduce direct healthcare emissions and reach healthcare decarbonisation goals such as the NHS England target to become ‘carbon net zero’ by 2040 [8].

Another concern associated with inhalational anaesthetic agents is occupational health risk, associated with long‐term exposure amongst healthcare staff. All inhalational anaesthetic agents are subject to regulation (e.g. Control of Substances Hazardous to Health (COSHH) in the UK), which means it is a legal requirement for employers to ensure good control practices and to establish reasonably practicable interventions if employee exposure exceeds the limit value [9, 10]. The assigned workplace exposure limit values in the UK are 50 parts per million (ppm) for isoflurane and 100 ppm for nitrous oxide, on the basis of an 8‐h time weighted average measurement [9]. Regulations governing design, installation and verification of medical gas pipeline systems confer adequate protection to most employees in hospital operating departments [11, 12]. In operating theatres, exposure is minimised using circle systems, anaesthetic gas scavenging systems and ventilation requirements, and is supported by education and awareness among anaesthetists. In addition, anaesthetists have agency over their practice and can elect to avoid inhalational anaesthetic agents almost completely, thereby minimising exposure for their colleagues as well as themselves.

However, anaesthetic gas scavenging system and operating theatre‐equivalent ventilation standards are not mandated in labour wards [13], even though recent data show that 76% of patients in the UK use nitrous oxide during labour [14]. Over a career, midwives and maternity support workers are therefore likely to have far higher cumulative exposure to nitrous oxide than anaesthetists, and yet awareness of the occupational hazards of nitrous oxide is not an integral component in midwifery education [15]. Instead, emphasis is placed on the widely accepted role of nitrous oxide as a safe analgesic and important component of non‐medicalised birth. Furthermore, the lack of widely accepted alternative labour analgesics means that intervention relies on nitrous oxide capture and destruction, rather than replacement.

From an environmental perspective, a study by Pearson et al. equated the ‘carbon footprint’ of 4 h intermittent use of nitrous oxide (i.e. for one patient using nitrous oxide only during contractions, occurring at a rate of three in 10 min) to driving 1400 km (237 kg of carbon dioxide equivalent (CO_2_e)) [16]. It follows that annual nitrous oxide use as labour analgesia in the UK contributes over 100 kT CO_2_e, comparable to driving 630 million km or nearly 16,000 trips around the equator [16].

Anaesthetic gas capture technology has so far focused on adsorption of volatile anaesthetic agents onto activated carbon or zeolite filters (known as vapour or volatile capture technology). Commercially available volatile capture technologies have been reviewed recently but none of these capture nitrous oxide [17]. It is, however, possible to install catalytic destruction technology, where a heated catalyst ‘cracks’ nitrous oxide into nitrogen and oxygen [18]. Such devices can be retrofitted to existing gas scavenging systems on a centralised basis or operated as mobile units at the point of care, for example in labour suite delivery rooms. According to one manufacturer, Medclair AB (Stockholm, Sweden), 99.6% of nitrous oxide can be removed using this approach, consistent with exposure monitoring data [18, 19].

Central destruction units, first introduced in 2004, are commonplace in Swedish hospitals. However, detailed life cycle assessment and life cycle cost analyses conducted by Stockholm County Council in 2011 report that multiple generations of central destruction units and progressive changes to operational set‐up were required to reach desirable efficiency. Installation was also facilitated greatly by pre‐existing nitrous oxide scavenging infrastructure and clinical practices, which are not implemented widely internationally, including in the UK [20]. Although the use of mobile destruction units avoids some of these issues, UK obstetric units that trialled the technology described barriers including a large equipment footprint and labour‐intensive set‐up [19]. As such, there is significant scope for improving the integration of such technologies into clinical practice.

By accepting that inhalational anaesthetic agents have an enduring role in anaesthesia and analgesia, there is an opportunity for innovative anaesthetic gas capture and destruction solutions to be impactful from both an environmental and occupational health perspective. To reduce anaesthetic gas emissions in the global health care industry effectively, sorbent material design should exhibit technical characteristics and target clinical needs (Table 1). Compared with industrial applications, candidate materials need to be effective for gas capture at very low partial pressures and to tolerate high humidity [21]. Ideal sorbents should capture all inhalational anaesthetic agents with high efficiency; support anaesthetic gas destruction or re‐use; and be reusable, economical and sustainable to produce. In recent years, an abundance of literature has been published on metal–organic frameworks (abbreviated as MOFs in the scientific literature), a novel class of sorbent materials which offers many desirable characteristics [22, 23, 24]. Although metal–organic frameworks have been investigated primarily for industrial gas capture, several studies have examined volatile anaesthetic agent uptake [25, 26, 27, 28], and one study investigated nitrous oxide capture in the context of anaesthesia [29].

**Table 1:** Key features to consider when designing sorbent materials for nitrous oxide capture.

Nitrous oxide sorbent key features	Details
Large gas storage ability and selectivity	Selectively target and store nitrous oxide with high efficiency, with an aim to reduce the cost of technologies in hospitals with fewer regenerations of the sorbent needed
Scalable, facile synthesis	For effective rollout of technology a viable manufacturing pipeline is needed, requiring a low‐cost sorbent with a simple, scalable synthesis and small carbon footprint
Low intensity regeneration	The sorbent must have a low intensity regeneration cycle, further reducing its recycling cost and carbon footprint. It should be easily regenerated and recyclable without losing uptake capacity and performance
High resistance to humidity	Exhaled breath has varying levels of relative humidity [21], therefore high resistance to humidity is needed to improve capture rates and longevity of the sorbent
Low cost	Synthesis and replacement of the sorbent should be cost effective for large‐scale rollout of the technology
Low environmental impact	In addition to using sustainable techniques to synthesise the sorbent, its waste disposal should be facile and have a small environmental impact

In this narrative review, we describe the main types of adsorbents with potential for clinical nitrous oxide capture, in terms of their porosity and chemistry, paying special attention to metal–organic frameworks. The way in which metal–organic framework materials can play a role in overcoming barriers presented by existing techniques and devices is also discussed.

## Methods

NHS England published ‘Delivering a ‘Net Zero’ National Health Service’ in 2020 [8]. To examine how developments in the field have progressed during the intervening time, a literature search of the last 5 years (1 January 2020–9 January 2025) was undertaken using the Clarivate Web of Science™ database. Search terms were combined with Boolean operators and included the following: ‘N_2_O capture’; ‘N_2_O sorbent’; ‘N_2_O activated carbon’; ‘N_2_O sorption’; ‘N_2_O zeolite’; ‘N_2_O metal–organic framework’; and ‘N_2_O MOF’. The search terms included both ‘nitrous oxide’ and ‘N_2_O’. Only original research articles written in English were included. Results were reviewed by abstract and articles were excluded if they concerned agricultural practices or nitrous oxide catalytic processes without reporting nitrous oxide uptake. Compiled data were converted to units of mmol.g^‐1^ and where tabulated/reported data were not available, data were read from graphs directly. For the discussed metal–organic frameworks, surface area data are given if available. The initial search retrieved 1025 articles, of which 43 were suitable for data analysis after abstract review and exclusion criteria were applied.

## Results

### Review of sorbent materials

The role of an adsorbent is to capture a molecule of interest through van der Waals and electrostatic interactions or chemical bonding. The mechanism of interaction is determined by the properties of the adsorbent itself, such as adsorbent geometry and surface chemistry. For gas capture applications, key considerations are the surface area and porosity of the material, and the selectivity of surface interactions with the target gas molecule. A key advantage of certain classes of porous adsorbents, that is, metal–organic frameworks, is that both the geometric and surface chemistry properties of their pores can be tuned for selective capture of a specific anaesthetic gas. A material with a larger surface area typically has more available sites for gas adsorption. However, the surface chemistry of the material is also important, which influences intermolecular bonding [30, 31]. Therefore, adsorbing large amounts of gas relies on both surface area and surface chemistry effects.

The three main classes of porous adsorbents, activated carbons, zeolites and metal–organic frameworks, are reviewed below. Their general structures and applications are described, as well as how they are already being implemented in anaesthetic gas capture technologies.

Activated carbons are derived from burning coal or biomass. Activation via a gas stream or chemical treatment functionalises carbons on the material surface, resulting in oxygen‐containing functional groups such as hydroxyls, carbonyls and carboxylic acids which can attract/bind molecules of interest [32]. They are highly porous, with surface areas ranging from approximately 250 m^2^.g^‐1^ to over 3500 m^2^.g^‐1^ [33, 34]. High chemical and thermal stability, low production cost and regenerative properties make activated carbons attractive adsorbents in many fields, from metal recovery to wastewater treatment [35]. They are the mainstay of current volatile capture technology for halogenated anaesthetic agents. They are used in both CONTRAfluran^®^ (Baxter/ZeoSys Medical GmbH, Luckenwalde, Germany) and SID‐Can (SageTech Medical, Paignton, UK) systems [36, 37]. Neither product is marketed for nitrous oxide capture.

**Figure 1:**
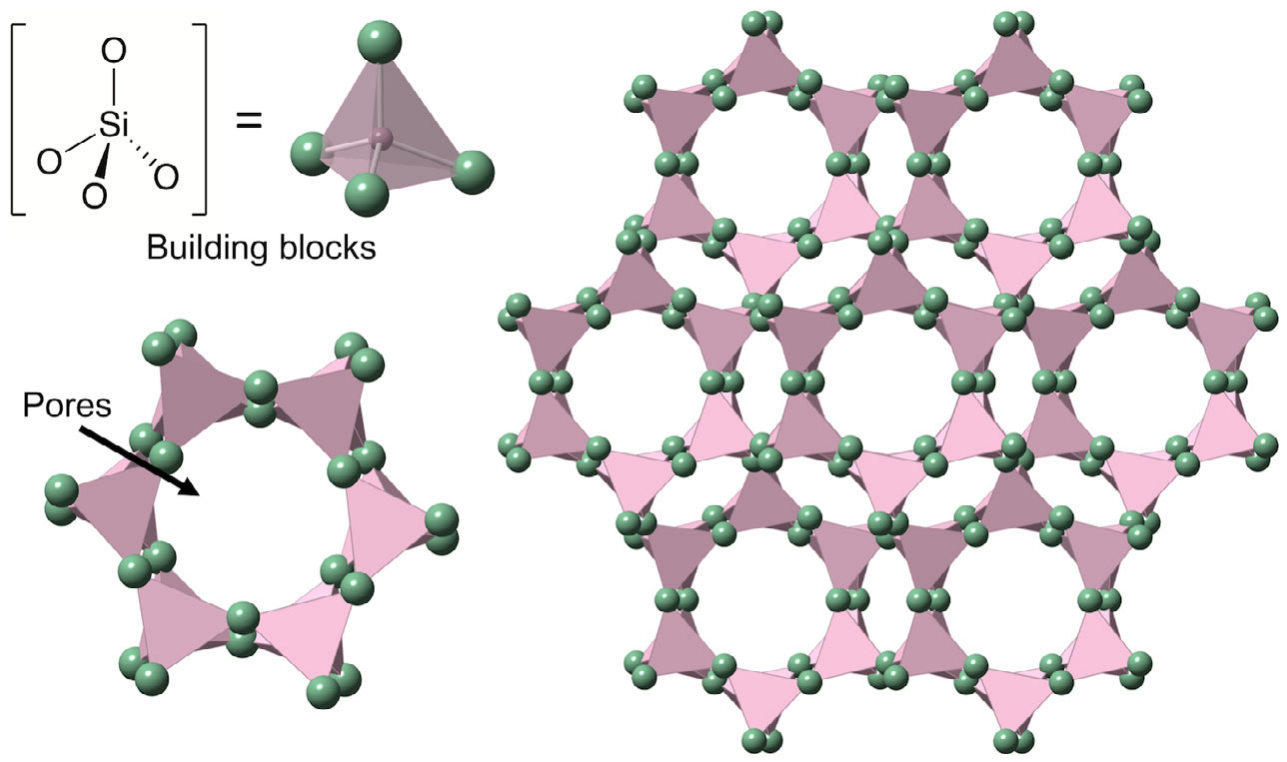
A representative Chabazite zeolite, showing the [SiO_4_]^4‐^ inorganic building blocks and the framework structure formed from [SiO_4_]^4‐^ tetrahedra linked by bridging oxygen atoms. Green, oxygen; pink, silicon. Image adapted from a CrystalMaker^®^ library file [43].

As shown in Fig. 1, zeolites are three‐dimensional, microporous crystalline structures consisting of an aluminosilicate framework of corner‐sharing tetrahedra, such as alumina and silica oxides ([AlO_4_]^5‐^ and [SiO_4_]^4‐^ groups), where each silicon and aluminium atom has four neighbours [38]. Typically, zeolites contain silicon and aluminium, but structural units comprising other elements are also possible, with oxygen atoms co‐ordinating to the metal ions [39]. The silica content of synthetic zeolites can be manipulated beyond that of natural zeolites, resulting in different pore shapes and distributions in the framework, giving a wider range of surface areas (approximately 10–1000 m^2^.g^‐1^) [38, 40].

Synthetic zeolites are important commercial adsorbents in petroleum refining and chemical process industries as catalysts, molecular sieves and ion exchangers [39]. Attractive qualities of zeolites include their industrial and large‐scale synthesis, and thermal and chemical stability [41]. At present, the only commercial volatile capture technology using zeolites is Deltasorb^®^ (Blue‐Zone Technologies, Ontario, Canada) [42].

**Figure 2:**
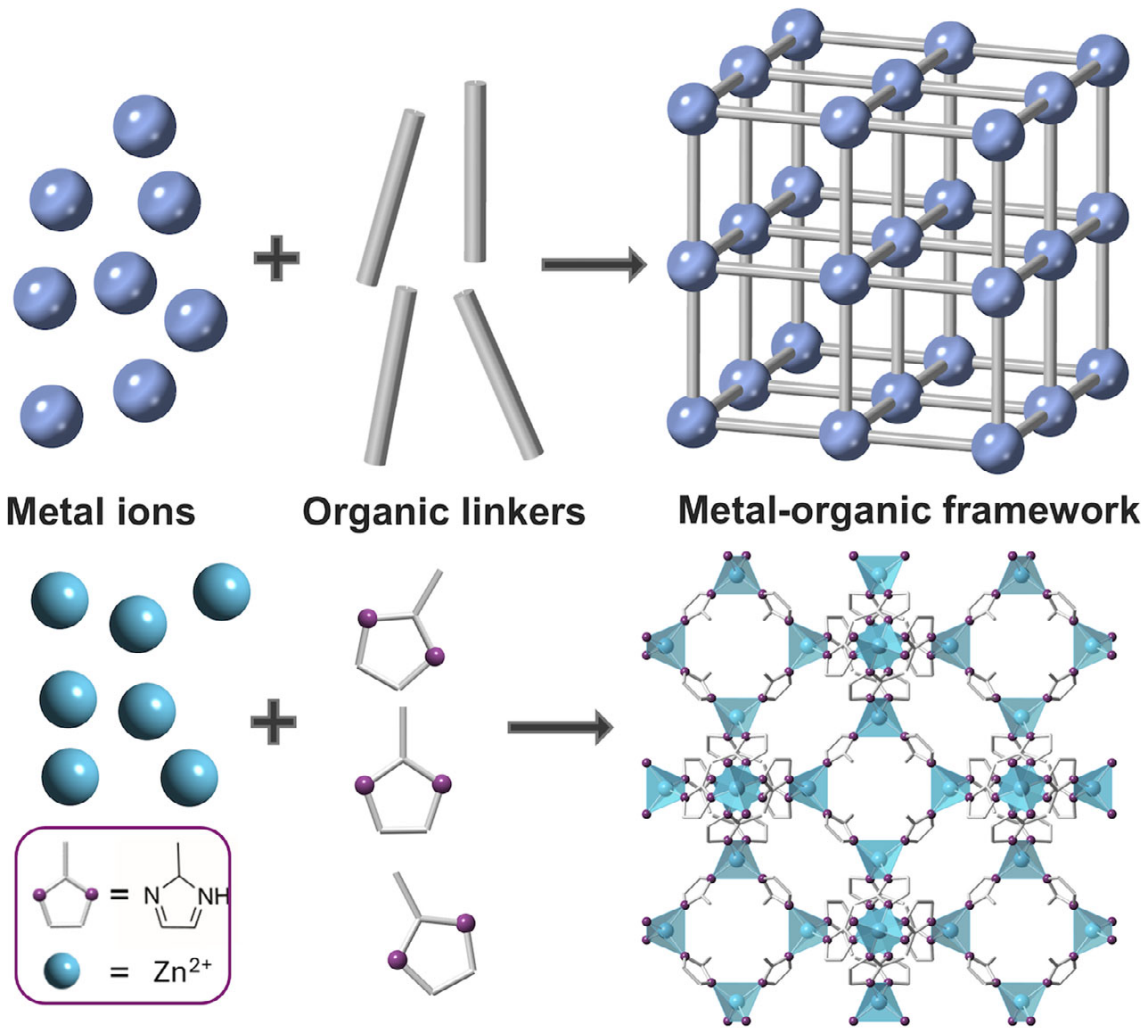
The self‐assembly of a metal–organic framework from metal ions and organic molecules to create repeating, porous structures (top). Representative example of the building blocks (Zn^2+^ ions and 2‐methylimidazole linkers) used to form a prototypical, commercially available metal–organic framework, ZIF‐8 (bottom). Zinc, blue; carbon, grey; nitrogen, purple. Hydrogen atoms have been omitted for clarity. Image created using CrystalMaker^®^ [43].

Metal–organic frameworks are another class of microporous materials, consisting of metal ions/clusters co‐ordinated to organic linker molecules (Fig. 2) [44]. The resulting structures are multi‐dimensional, highly porous frameworks which can trap specific gases [27, 45]. Metal–organic frameworks are modular in structure; each molecular building block can be exchanged with thousands of alternatives, offering more structural variability than activated carbons and zeolites. Selecting specific metal ions and linker molecules imparts different structural and chemical properties on the metal–organic framework and can, for example, significantly improve the hydrophobicity of the material, which is a key advantage over other adsorbents that suffer from water saturation [46, 47]. Moreover, selecting specific building blocks directly affects the pore size and surface chemistry of the structures, meaning metal–organic frameworks can be tailored for targeted uptake and enhanced selectivity for a particular molecule in gas mixtures.

Due to these properties, metal–organic frameworks have shown outstanding potential in numerous gas uptake and storage applications [48]. Their high internal surface areas (1000–7800 m^2^.g^‐1^) are larger than those of activated carbons and zeolites [49], and 1 g of some metal–organic frameworks materials exceeds the surface area of a football field (6400 m^2^) [50]. Another key feature of metal–organic frameworks is their ability to be regenerated over multiple cycles of sorption/desorption without significant compromise to their uptake capacity [51, 52]. Over 100,000 different metal–organic frameworks have been synthesised already, with additional hypothetical structures generated computationally, giving researchers a plethora of metal–organic frameworks to investigate for bespoke gas adsorption applications [53]. Despite their potential, there are no commercially available metal–organic frameworks presently in use in anaesthesia or with a dedicated clinical application.

### Nitrous oxide pressure scenarios for gas capture

Beyond physical characteristics, another consideration affecting the performance of an adsorbent is the conditions in which it is expected to operate. This requires knowledge of the partial pressure of a chosen gas, usually in the context of a gas mixture, which the adsorbent is intended to capture. Naturally, these partial pressures vary considerably across the wide range of industries in which adsorbents are used.

**Figure 3:**
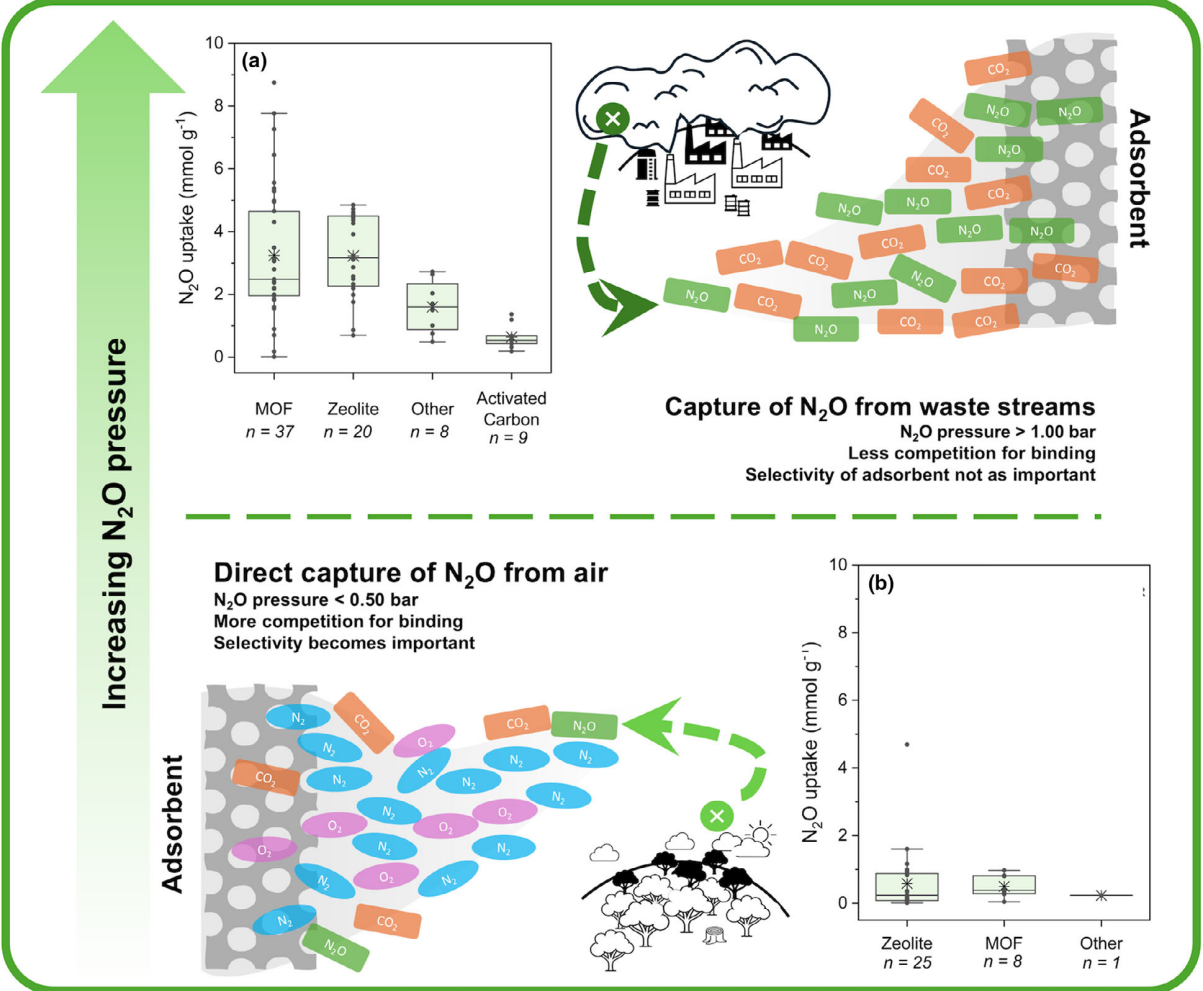
Nitrous oxide capture in different sorbent materials shown for two applications. The top figure shows capture from tail gas with (a) recent literature data as a box‐and‐whisker plot from nitrous oxide pressures > 1 bar. The bottom figure shows capture from the atmosphere with (b) recent literature data as a box‐and‐whisker plot for low pressure nitrous oxide experiments of < 0.5 bar. Box‐and‐whisker plots show: box, interquartile range; line within box, median value; ●, individual data point; ✷, mean value; whiskers, 5th and 95th percentile; n, number of adsorbents reported. Data selected from experiments performed at 25–30°C, no reported data found for nitrous oxide pressures between 0.5 and 1 bar [34, 38, 41, 54, 55, 56, 57, 58, 59, 60, 61, 62, 63, 64, 65, 66, 67, 68, 69, 70, 71, 72, 73, 74, 75, 76]. MOF, metal–organic framework; N_2_O, nitrous oxide.

Figure 3 compares recent experimental data from 26 studies comparing nitrous oxide capture by the different classes of porous adsorbents [34, 38, 41, 54, 55, 56, 57, 58, 59, 60, 61, 62, 63, 64, 65, 66, 67, 68, 69, 70, 71, 72, 73, 74, 75, 76]. Box‐and‐whisker plots have been used to display the collated data sets, showing wide variations in nitrous oxide uptake between, and within, different classes of adsorbent. The results of these studies are separated into two pressure boundaries: studies where sorption was tested/measured in scenarios with high partial pressures of nitrous oxide (≥ 1 bar); and scenarios with low partial pressures of nitrous oxide (< 0.5 bar). Most studies evaluated here relate to the former, examples of which include industrial processing of exhaust gas from large‐scale chemical reactions (tail gas) [34, 54, 55, 56, 57, 58, 59, 60, 61, 62]. By comparison, the partial pressures of exhaled nitrous oxide from a demand valve mouthpiece or anaesthetic breathing circuit and/or direct capture from ambient air in the anaesthetic or delivery room are approximately 0.5 bar and 0.0003 bar respectively at room temperature. These partial pressures are far lower than is relevant to industrial scale nitrous oxide capture, where the bulk of literature is focused.

Collectively, these studies show that most adsorbents analysed perform better in high‐pressure scenarios, with nitrous oxide sorption values approaching 9 mmol.g^‐1^ for some metal–organic frameworks (the uptake for a carbon dioxide adsorbent at 0.15 bar and room temperature is typically within the range of 0.2–6.0 mmol.g^‐1^) [48, 77]. However, at low, clinically relevant partial pressures, where competition between gases becomes more significant, nitrous oxide uptake falls below 2 mmol.g^‐1^, even for the best‐performing adsorbents.

As the global warming potential of nitrous oxide has become more widely appreciated, there has been increased motivation to study its capture at low partial pressures, using the principles of direct air capture from the ambient environment. Since nitrous oxide and carbon dioxide have very similar properties (linear molecules with the same molecular weight, kinetic diameter and boiling points within 10°C [62]), development of such technology has focused on the capture of low levels (approximately 420 ppm) of carbon dioxide from the atmosphere [78, 79]. While this means that sorbents that capture carbon dioxide successfully should be applicable to nitrous oxide capture, designing materials to be selective for nitrous oxide over carbon dioxide at such low pressures poses difficulties.

Overall, the potential to capture nitrous oxide at such low partial pressures is an area yet to be explored thoroughly; at < 0.5 bar, the average nitrous oxide uptake for metal–organic frameworks is comparable with that of zeolites (Fig. 3). However, the generation of computational libraries of metal–organic frameworks has provided researchers with a unique opportunity to design new sorbent materials with specific characteristics to meet these clinical needs [53, 80, 81, 82]. Such materials could be tailored to bind strongly and with high selectivity to nitrous oxide, therefore ensuring efficient uptake in the presence of other gases, for example, carbon dioxide, oxygen, water vapour and in low nitrous oxide partial pressure conditions.

### State‐of‐the‐art nitrous oxide adsorbents

Both activated carbons and zeolites have been commercialised for the capture of waste anaesthetic gas [36, 42]. However, filters incorporating activated carbons and zeolites have several limitations. The commercialisation of metal–organic frameworks for clinical anaesthetic applications has not been realised, but is a promising avenue for meeting the perquisites for an efficient sorbent material for waste anaesthetic gas capture.

While activated carbons are cheap to produce, little work has been undertaken in recent years to apply them to nitrous oxide capture from gas streams. The pore sizes and surface area of activated carbons underpin their adsorption capacity; pores approximately double the molecular diameter of nitrous oxide determine optimal uptake [34], while larger pores can allow condensation of water molecules which leads to poor adsorption performance of small gas molecules [83]. In addition, activated carbons suffer from reduced adsorption performance in the presence of relative humidity [83, 84]. At high relative humidity, however, the presence of water can be beneficial for gas uptake in some cases, but this has not been investigated for nitrous oxide [85].

While tuning the structures of activated carbons is limited mainly to thermal methods, activated carbons can be chemically functionalised through various routes, such as oxidation with acids and nitrogen‐doping with strong bases [86, 87]. However, research into chemical modifications of activated carbons to promote nitrous oxide adsorption is lacking. In some cases, modifying the structures of activated carbons can decrease selectivity for nitrous oxide; one example showed that altering the functionality of an activated carbon through treatment with a base reduced the uptake of nitrous oxide over carbon dioxide [56].

Microporous zeolites are suitable for gas capture as they exhibit high capacities even at small gas pressures because the gases are absorbed into their micropores [88]. They display comparatively higher uptake than activated carbon adsorbents because of these stronger interactions between the gas molecules and the micropores of zeolites [88]. Their uptake capacity is also linked to their aluminium content, where zeolites containing a higher amount of aluminium display higher uptakes; designing zeolites with a higher aluminium content are thus attractive for many applications [88]. Zeolites, such as zeolite 13X and Zeolite Socony Mobil–5, have been studied for halogenated anaesthetic agent capture and can outperform activated carbons in these applications [45, 89, 90, 91]. In addition, several zeolites have been investigated for nitrous oxide adsorption [62]. A benefit of zeolites is that their compositions can be chemically tuned to boost uptake of nitrous oxide and selectivity over carbon dioxide by substituting metal ions in the structure [41, 55]. This ability allows zeolites to outperform silica gel and activated carbons for uptake even at trace amounts of nitrous oxide [38, 70, 92].

However, at high humidity, the performance of zeolites is significantly diminished [85, 93]. This has been observed in a zeolite 13X derivative, where water molecules displaced isoflurane molecules resulting in lower isoflurane uptake [90]. This can be mitigated by reducing the hydrophilic aluminium content in zeolites; however, as increased aluminium content improves nitrous oxide uptake, this is a delicate balance [45]. Additionally, although selectivity to nitrous oxide can be tuned, many zeolite structures exhibit decreased nitrous oxide adsorption in the presence of carbon dioxide [38].

### Metal–organic frameworks

Although metal–organic frameworks have several attractive properties compared with alternative adsorbents, their structural tunability for precision adsorption of a target gas confers the most versatility. The modular architecture of metal–organic frameworks means this tunability can be achieved by alterations in numerous different structural components, and this has been shown to have a significant impact on functionality. As with zeolites, most studies referenced here report nitrous oxide adsorption on metal–organic frameworks at higher nitrous oxide partial pressures than those relevant for medical applications, including nitrous oxide/carbon dioxide separations (Table 2) [59, 73], but these are included for illustrative purposes.

**Table 2:** Properties of select metal–organic framework from literature, including nitrous oxide uptake, surface area and experimental conditions (all experiments were performed at atmospheric conditions and 25°C). Definitions of metal–organic framework structures and linkers can be found in online Supporting Information Appendix S1.

Metal–organic framework	Modification	Nitrous oxide pressure; bar	Nitrous oxide uptake; mmol.g^‐1^	Surface area*; m^2^.g^‐1^	Ref.
(1) Ce_TzTz	‐	1.00	2.20	1136.0	[70]
(2) Ce_TzTz_PyPy	Second linker introduced	1.00	0.70	283.0	[70]
(3) Zn(hba)	‐	1.00	2.48	Unreported in reference	[63]
(4) Zn(2‐Mehba)	Two methyl groups introduced	1.00	0.18	Unreported in reference	[63]
(5) MIL‐101‐NO_3_	‐	1.00	2.37	3915.0	[75]
(6) MIL‐101‐Cl	Stronger electron withdrawing group	1.00	5.56	4224.0	[75]
(7) Co_2_(dobdc)	‐	1.00	7.26	Unreported in reference	[69]
(8) Mg_2_(dobdc)	Co^2+^ exchanged for Mg^2+^	1.00	8.75	Unreported in reference	[69]
(9) DMOF‐M	‐	0.10	0.30	1679.9	[57]
(10) DMOF‐3 M	High polarity via more methyl groups	0.10	0.80	1205.6	[57]
(11) MIL‐126(Sc)	‐	0.01	0.04	1428.0	[76]
(12) MIL‐126(Cr/Sc)	Additional metal centre	0.01	0.27	1354.0	[76]

* Calculated using Brunauer–Emmett–Teller theory [92].

One study investigated reducing the nitrous oxide sorption capacity in favour of carbon dioxide by adding an additional linker to a cerium‐based metal–organic framework (1, Table 2). The resulting metal–organic framework (2, Table 2) had a smaller surface area, with a decreased nitrous oxide capacity (2.20 to 0.70 mmol.g^‐1^). The unmodified metal–organic framework (1, Table 2) adsorbed nitrous oxide preferentially, whereas the modified analogue (2, Table 2) favoured the adsorption of carbon dioxide [70]. The pore sizes of metal–organic frameworks can also be tuned to improve selectivity; this has also been exploited to reduce nitrous oxide selectivity over carbon dioxide. For example, large square channels of 0.6 × 0.6 nm present in a zinc‐based metal–organic framework (3, Table 2) could accommodate large amounts of carbon dioxide and nitrous oxide with little selectivity, while the introduction of two methyl groups to create a modified metal–organic framework (4, Table 2) exhibited reduced nitrous oxide uptake from 2.48 to 0.18 mmol.g^‐1^ [63].

Research has also shown that uncoordinated metal sites in metal–organic frameworks can interact with gas molecules allowing strong adsorption and facilitating selectivity [69]. Furthermore, metal–organic framework architectures can be designed synthetically to chemically tailor a metal site for efficient interactions with target gases. Zhang et al. exploited this concept on a chromium‐based metal–organic framework series (5 and 6, Table 2) showing that the introduction of Cl‐ ions increased the number of binding sites for nitrous oxide and increased uptake from 2.37 mmol.g^‐1^ to 5.56 mmol.g^‐1^ [75]. Altering the metal centre itself in a metal–organic framework can also increase uptake; Pitt et al. studied the uptake of structurally similar metal–organic frameworks (7 and 8, Table 2) with different metal ions and showed that the magnesium‐containing analogue exhibited the highest uptake (8.75 mmol.g^‐1^) [69].

The polarity of metal–organic framework adsorbents can also be manipulated to selectively adsorb more polar molecules (e.g. nitrous oxide) over nonpolar molecules such as molecular nitrogen. Wang et al. showed that introducing methyl groups into a nickel‐based metal–organic framework structure (9, Table 2) increased its nitrous oxide capacity at low pressures. Selectivity for nitrous oxide over molecular nitrogen in the methylated metal–organic framework (10, Table 2) also increased [57].

**Figure 4:**
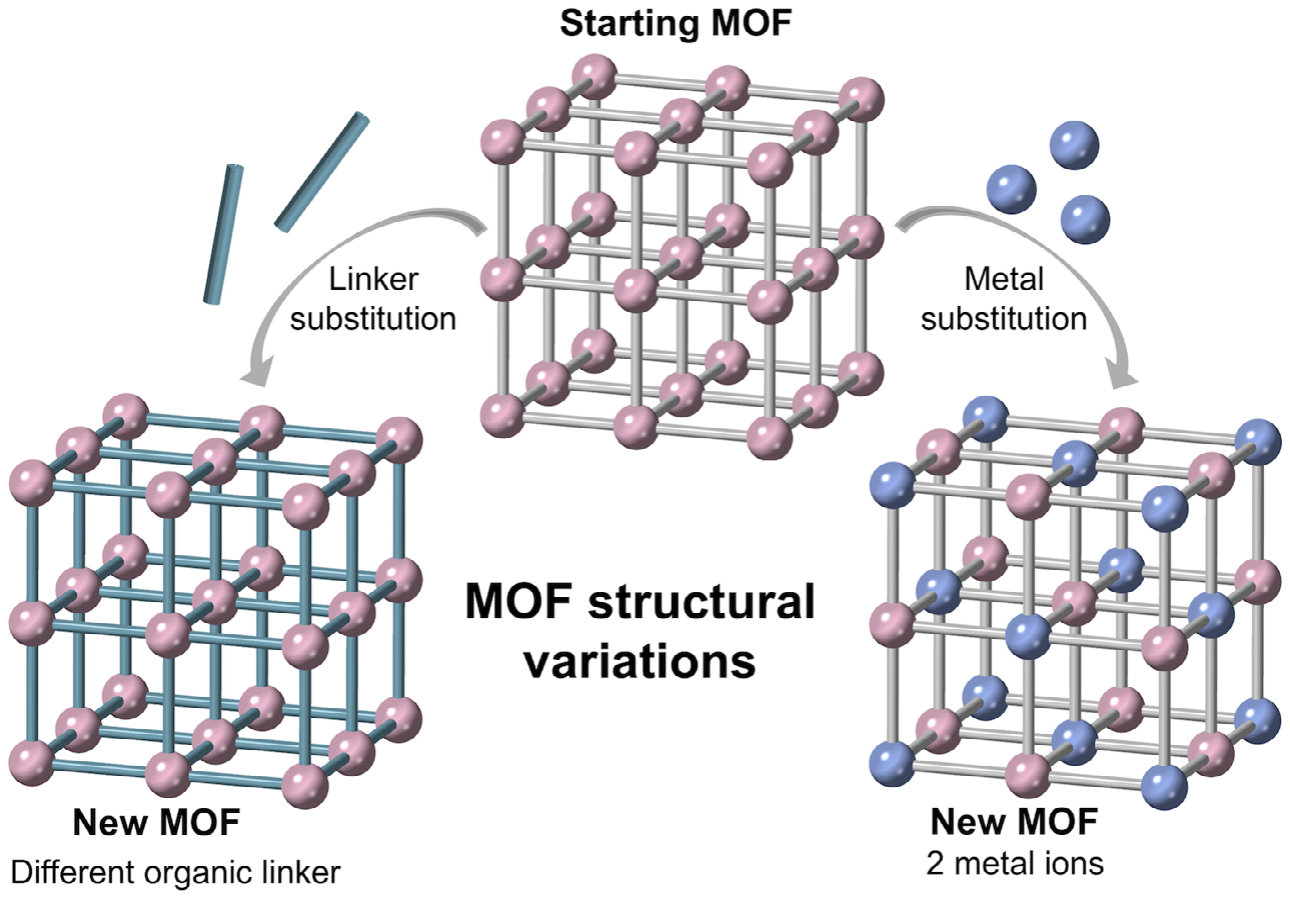
Possible structural modifications to an existing metal–organic framework (MOF) structure to produce new metal–organic framework structures with different properties.

Another attractive quality of metal–organic frameworks is that their structures can be altered post‐synthesis (Fig. 4), where the organic linker can be changed chemically and metal ions can be introduced and substituted into the structures [76, 94].

Few studies have investigated the nitrous oxide adsorption performance of metal–organic frameworks at low partial pressures [57, 66, 76, 95]. One study looked at a metal–organic framework containing two different metal ions; these metal–organic frameworks are of interest because of the greater diversity of chemical characteristics that are introduced with the addition of each metal (Fig. 4). A bimetallic metal–organic framework (12, Table 2) containing Cr^3+^ and Sr^3+^ ions displayed enhanced nitrous oxide uptake at low nitrous oxide partial pressures (0.01 bar at 25°C) relative to the metal–organic framework (11, Table 2) containing only Sr^3+^. Stronger interactions between nitrous oxide and Cr^3+^ ions vs. Sc^3+^ ions resulted in sorption values of approximately 0.27 mmol.g^‐1^ and 0.04 mmol.g^‐1^ for the bimetallic metal–organic framework (11, Table 2) and metal–organic framework (12, Table 2), respectively [76].

Although zeolites and metal–organic frameworks can be structurally tuned, more possible structural variations for metal–organic frameworks have resulted in a much higher number of identified metal–organic frameworks (circa 100,000 vs. > 250 zeolites) [96]. The structural variability of metal–organic frameworks gives researchers more potential candidates for nitrous oxide capture and provides more opportunities for chemical fine‐tuning to boost adsorption performance. This is especially attractive because computational researchers can screen metal–organic framework databases for top structures and simulations can predict which structures will exhibit the best adsorption performance for bespoke applications; this has been used extensively for predicting top metal–organic framework candidates for carbon dioxide capture [53, 80, 81, 82]. For nitrous oxide capture specifically, metal–organic framework structures can be screened for high nitrous oxide uptake at relevant partial pressures down to the ppm range, in the presence of other gases such as carbon dioxide and water vapour [72, 97]. From an anaesthetic perspective, these metal–organic frameworks would have applications in environments where an anaesthetic gas scavenging system is not available. Direct air capture for carbon dioxide and other gases has already shown promise and could be extended to nitrous oxide capture [98, 99].

Life cycle analysis of any new technology is important for environmental and economic viability. Researchers are actively searching for new facile and green synthetic procedures to fabricate metal–organic frameworks and metal–organic framework‐derived materials, moving away from traditional costly syntheses requiring relatively large quantities of solvent [100]. Advances in efficient scale‐up of metal–organic frameworks on an industrial scale are underway and an increasing number of global organisations are investing in metal–organic framework technology [101, 102]. Moreover, metal–organic frameworks can be reused in multiple adsorption cycles because of their facile regeneration [52].

### The future of metal–organic frameworks in anaesthesia

**Figure 5:**
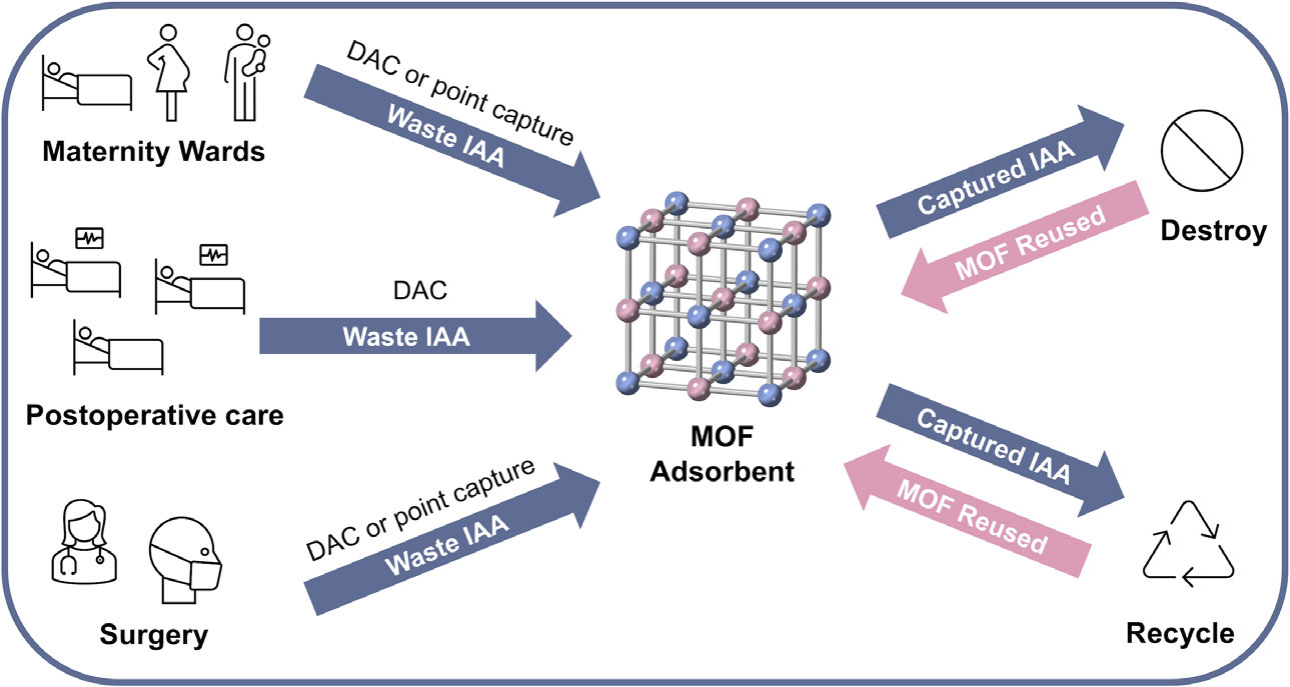
The use of metal–organic frameworks (MOF) for capturing waste inhalational anaesthetic agents in various anaesthesia applications. Metal‐organic frameworks can be implemented to capture inhalational anaesthetic agents (IAA) via point source capture from anaesthetic breathing circuits or direct air capture (DAC) from rooms lacking extraction. The adsorbed inhalational anaesthetic agents could then be destroyed or recycled, and the metal–organic framework reused.

There is a lot of promise for metal–organic frameworks in novel gas capture applications such as anaesthesia. The progress of research in metal–organic framework applications at low gas partial pressures indicates that the complex nature of exhaled gas streams containing nitrous oxide can be targeted effectively. By selecting their building blocks carefully, metal–organic frameworks can be designed to overcome humidity issues encountered by many adsorbent materials and selectively adsorb inhalational anaesthetic agents of interest within a gas mixture, and in the presence of carbon dioxide. This technology could be used in numerous contexts spanning anaesthetic practices. Different metal–organic framework adsorbents tailored for the capture of specific anaesthetic gas or nitrous oxide at low gas partial pressures could be used for direct air capture in the anaesthetic room, postoperative care facility, labour suite delivery rooms or any remote site where an anaesthetic gas scavenging system is not available. Metal–organic frameworks tailored for higher gas partial pressures could be incorporated into the expiratory limb of a demand valve mouthpiece, facilitating point of use scavenging of nitrous oxide in delivery suites, clinics or even in ambulatory settings (i.e. ambulances), since no power source is required (Fig. 5).

Wide adoption of metal–organic framework technology for these applications could reduce the impact of anaesthetic practice on global warming dramatically and reduce staff exposure in a wide range of settings. Within anaesthesia, nitrous oxide currently contributes by far the largest environmental harm [1], and several favourable properties of metal–organic frameworks make them particularly promising candidates as successful nitrous oxide sorbents (Table 3), where they meet key requirements outlined in Table 1.

**Table 3:** Key features of a successful nitrous oxide sorbent, as described in Table 1, and how metal–organic frameworks can meet these challenges.

Nitrous oxide sorbent key features	Metal–organic framework property
Large gas storage ability and selectivity	Metal–organic frameworks can adsorb high amounts of nitrous oxide at low partial pressure conditions, with many modifications identified to encourage selectivity over other gases [57, 67]
Scalable, facile synthesis	The scale up of metal–organic framework production is already being tackled by global industry and academia [101]
Low intensity regeneration	Metal–organic frameworks show good regeneration properties, allowing multiple cycles of sorption and desorption. Work is ongoing to ensure this can be done under milder conditions [101]
High resistance to humidity	Metal–organic frameworks can be designed to have high resistance to humidity in comparison to zeolites and activated carbons [46, 47]
Low cost	Computational studies have streamlined the metal–organic framework discovery process and will undoubtedly contribute to identifying low‐cost metal–organic frameworks with high performance [72, 97]. Research into cost‐efficient synthetic routes is underway [78, 104, 105]
Low environmental impact	Green synthesis of metal–organic frameworks is an active area of research, with greener syntheses routes under investigation [100, 102, 103]

Metal–organic frameworks do not always alter the chemical structure of the chemicals they capture, such that, combined with new or existing systems, anaesthetic gas or nitrous oxide could be desorbed in a controlled environment and destroyed or processed for reuse (in accordance with regulatory permissions). The result would be to regenerate the metal–organic framework for further use and thereby create a complete and sustainable system for anaesthetic gas removal.

Due to the success in metal–organic framework research for carbon dioxide capture, there is already infrastructure in place for large‐scale metal–organic framework manufacture [101, 106]. Commercial metal–organic frameworks are already available from multinational companies, and smaller companies are rolling out pilot‐scale metal–organic framework manufacturing projects [107, 108]. Metal–organic framework technology is becoming increasingly accessible and there is additional research investigating the carbon footprint of metal–organic framework manufacture [109]. These are important attributes to develop a technology that improves on the existing commercial offerings. As recently as 2024, volatile capture technology has been rejected for use in NHS Scotland for varied reasons including the lack of life cycle assessments and the uncertainties around the efficiencies of the capture technologies evaluated [110].

The introduction of metal–organic framework adsorbents into the NHS has the promise of helping institutions reach the ‘net zero’ targets of reduced greenhouse emissions. More importantly, these fascinating structures could provide a realistic solution to a long‐overlooked area within the NHS: the prolonged exposure of maternity staff to potentially harmful nitrous oxide. With metal–organic frameworks designed on the principle of direct air capture, adsorbents can be implemented to uptake trace amounts of nitrous oxide in maternity wards, and indeed other facilities which lack extraction infrastructure, giving scope for metal–organic frameworks to have a significant role in anaesthetic scavenging in low‐resource settings. This itself warrants further investigation of these materials and the systems that could be used to facilitate their introduction into hospitals.

## Supporting information


**Appendix S1.** Abbreviations and definitions relating to Table 2 in the main document.
