# Supplementary material for: Developments in nitrous oxide capture technologies: bridging current research to clinical applications
Source: Anaesthesia. 2025 Dec 3;81(3):415–29. doi: 10.1111/anae.70080 (PMC12893838; doi:10.1111/anae.70080)
Supplement: Supplementary file 1 — Appendix S1. Abbreviations and definitions relating to Table 2 in the main document. [file ANAE-81-415-s001.docx]

**Appendix S1** Abbreviations and definitions relating to Table 2 in the main document

| Ce_TzTz | [Ce_6_O_4_(OH)_4_(TzTz)_6_], H_2_TzTz = [2,2′-bithiazole]-5,5′-dicarboxylic acid |
| --- | --- |
| Ce_TzTz_PyPy | [Ce_6_O_4_(OH)_4_(TzTz)_4_(PyPy)_2_], H_2_TzTz = [2,2′-bithiazole]-5,5′-dicarboxylic acid, H_2_PyPy = 2,2′-bipyridine-5,5′-dicarboxylic acid) |
| Zn(hba) | H_2_hba = 4-hydroxybenzoicacid |
| Zn(2-Mehba) | 2-Mehba^2-^ = 2-methyl-4-hydroxybenzoic acid |
| MIL-101-NO_3_ | [Cr_3_(O)NO_3_(bdc)_3_ (H2 O)_2_]·nH_2_O, (bdc=benzene-1,4-Dicarboxylate) |
| MIL-101-Cl | [Cr_3_(O)Cl(bdc)_3_ (H2 O)_2_]·nH_2_O, (bdc=benzene-1,4-Dicarboxylate) |
| Co_2_(dobdc) | dobdc^4-^ = 2,5-dioxido-1,4-benzenedicarboxylate |
| Mg_2_(dobdc) | dobdc^4-^ = 2,5-dioxido-1,4-benzenedicarboxylate |
| DMOF-M | (Ni_2_(BDC-M)_2_DABCO), BDC-M = monomethyl 1,4-benzenedicarboxylate/terephthalate, DABCO = , 4-diazabicyclo-[2,2,2] octane |
| DMOF-3M | (Ni_2_(BDC-TM)_2_DABCO), BDC-M = ,3,5,6-tetra-methyl 1,4-benzenedicarboxylate/terephthalate, DABCO = , 4-diazabicyclo-[2,2,2] octane |
| MIL-126(Sc) | [Sc_3_-O(H_2_O)_2_(bpdc)_3_X]_n,_ bpdc = biphenyl-4,40-dicarboxylate , X = OH^-^ or Cl^-^ |
| MIL-126(Cr/Sc) | [(Cr/Sc)_3_-O(H_2_O)_2_(bpdc)_3_X]_n,_ bpdc = biphenyl-4,40-dicarboxylate , X = OH^-^ or Cl^-^ |
